# A Miniaturized On-Chip Colorimeter for Detecting NPK Elements

**DOI:** 10.3390/s16081234

**Published:** 2016-08-04

**Authors:** Rui-Tao Liu, Lu-Qi Tao, Bo Liu, Xiang-Guang Tian, Mohammad Ali Mohammad, Yi Yang, Tian-Ling Ren

**Affiliations:** 1 Institute of Microelectronics, Tsinghua University, Beijing 100084, China; lrt16@mails.tsinghua.edu.cn (R.-T.L.); taoluqi@126.com (L.-Q.T.); electroniclover@hotmail.com (B.L.); txg19941015@163.com (X.-G.T.); mam20@ualberta.ca (M.A.M.); yiyang@tsinghua.edu.cn (Y.Y.); 2 Tsinghua National Laboratory for Information Science and Technology (TNList), Tsinghua University, Beijing 100084, China; 3 School of Chemical and Materials Engineering (SCME), National University of Sciences and Technology (NUST), Sector H-12, Islamabad 44000, Pakistan

**Keywords:** colorimeter, NPK elements, Beer-Lambert’s Law

## Abstract

Recently, precision agriculture has become a globally attractive topic. As one of the most important factors, the soil nutrients play an important role in estimating the development of precision agriculture. Detecting the content of nitrogen, phosphorus and potassium (NPK) elements more efficiently is one of the key issues. In this paper, a novel chip-level colorimeter was fabricated to detect the NPK elements for the first time. A light source–microchannel photodetector in a sandwich structure was designed to realize on-chip detection. Compared with a commercial colorimeter, all key parts are based on MEMS (Micro-Electro-Mechanical System) technology so that the volume of this on-chip colorimeter can be minimized. Besides, less error and high precision are achieved. The cost of this colorimeter is two orders of magnitude less than that of a commercial one. All these advantages enable a low-cost and high-precision sensing operation in a monitoring network. The colorimeter developed herein has bright prospects for environmental and biological applications.

## 1. Introduction

Around 2005, a farming management concept known as precision agriculture had been put forward. According to this concept, various parameters such as the concentration of soil nutrients, consumption of water, and air temperature are measured in order to optimize the growth environment of crops [1,2,3,4]. Crop growth is related to humidity, air temperature, soil nutrients (concentration of NPK (Nitrogen-Phosphorus-Potassium) elements), etc. In China, some soil NPK detection results [5,6] have been published by the government in different provinces and measurement standards [7,8] have been set. Meanwhile, some scientific groups have tried to detect the concentration of nutrition elements in soil based on basic electro-chemical methods. K.A. Sudduth and colleagues [9] have detected some major elements of soil nutrients by measuring the changes in the conductivity of soil samples. Adamchuka et al. [10,11] directly measured soil chemical properties by using ion-selective electrodes. Black [12] and Lu [13] mention more detailed methods to detect the concentrations of both common elements and rare elements. Meanwhile, commercial detectors based on colorimetry have already been widely used in some analysis fields [14,15,16,17]. Some researchers summarized the detection methods and compared them [18,19,20,21]. However, most detectors mentioned above are limited to large-volume sensors, complex chemical detection methods or experimental conditions. In this paper, we build a miniaturized detection system based on Beer-Lambert’s law and MEMS technology which differs from any other previous work.

Beer-Lambert’s law relates the attenuation of light to the properties of the material through which the light is traveling. This law is commonly applied to chemical analysis measurements and light calibrations; for example, Uludag and Kamil [22] estimated the near-infrared wavelengths and Andrei A. Bunaciu, Hassan Y. Aboul-Enein, Serban Fleschin [23] analyzed pharmaceutical drugs using the law. Besides, by combining this law with some modern analytical theory and tools, the modified Beer-Lambert’s law has been more widely used in new analysis fields. According to analysis reflectance and transmittance spectroscopy, some functions of the human brain, skin color and some other biological information can be easily detected in a non-invasive way [24,25,26]. This law is also expected to be widely used in new material microstructure analysis [27,28,29].

In this paper, a novel chip-level colorimeter was fabricated to detect nutrition elements in soil based on Beer-Lambert’s law. Compared with commercial colorimeters, the volume used in our device can be very small because of MEMS technology. Besides, the advantages of low cost, fewer testing samples and high precision are achieved.

## 2. Experimental Section

### 2.1. Theory of Beer-Lambert’s Law

The basic detection mechanism of our sensor is Beer-Lambert’s law. Beer-Lambert’s law is a traditional attenuation theory of light, and it can be applied to detect concentrations of certain mixed liquids. Furthermore, it has a linear relationship between attenuation and the concentration of the analyte.

There are some conditions that need to be fulfilled in order for Beer-Lambert’s law to be valid. These conditions are:

(1) The incident radiation must consist of parallel rays and preferably be monochromatic.
(2) The attenuating medium must be homogeneous and not scatter the radiation.
(3) The attenuators must act independently of each other.
(4) The incident radiation must not influence the atoms or molecules. There must only be light absorption but no fluorescence generation or photochemical reactions are allowed.

Beer-Lambert’s law is given by:

(1) $$A=-log_{10}\frac{I_{t}}{I_{0}}=log_{10}\frac{1}{T}=K\cdot l\cdot C$$

where *A* is the absorbance of the sample, *I*_0_ is the incident light intensity, it is the transmitted light intensity, *T* is the transmittance (*T* = *I*_0_*/I_t_*), *K* is the attenuation coefficient, *l* is the optical depth, and *C* is the concentration of the sample. We can set *K* as a constant and want to achieve larger changes of *A* when the concentration of the sample has small changes, so *l* is a parameter which has a relationship with the sensitivity of the device. Before we fabricate the sensor, the absorption spectrum of the analyte should be tested first. We select the highest absorption waveband as the light source in order to maintain a high absorption rate when the sample concentration is very low, and the more the absorption light intensity changes, the greater detection accuracy will be achieved. Therefore, for our experiment, the nitrogen test solution looks light blue, the phosphorus test solution is colorless, and the potassium test solution exhibits turbidity. After absorption spectrum analysis, a red light is selected as the incident radiation to detect both nitrogen and phosphorus, and a blue light will be used to detect potassium.

### 2.2. System Components

**Figure 1:**
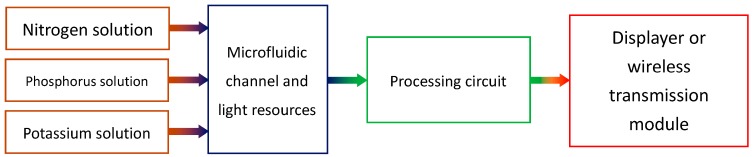
Colorimeter system components.

The whole colorimeter system consists of some components, which are shown in Figure 1. The three major sub-systems include the sensors, the circuitry, and the display or transmission module.

#### 2.2.1. Micro-Fluidic Channel and Light Resources

**Figure 2:**
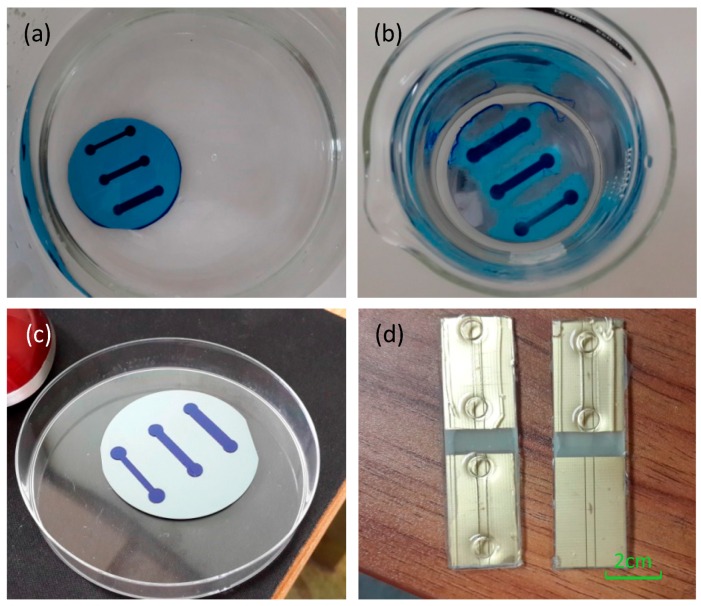
Fabrication of microchannel. The development procession at (**a**) 0 min; and (**b**) after 20 min; Panel (**c**) shows three channels (1 mm, 2 mm and 3 mm); Panel (**d**) shows microchannels.

The micro-fluidic channel is fabricated using soft-lithography processes and consists of the channel with a length of 2 cm, a height of 300 μm and a width between 2 mm to 6 mm. Three different channel widths are designed in order to compare the results due to different conditions (flow rate, pressure, etc.). The ratio of the PDMS (polydimethylsiloxane) and the hardener mixture is different between the upper layer (1:10) and the protection layer (1:5) in order to make a more stable combination. The procession to fabricate the micro-channel is shown in Figure 2.

In the experiment, the red and blue on-chip light sources are used in order to match the sandwich structure of the micro-fluidic channel. Both of the light sources are on the order of microns and the emission wavelength range of the red light (ES-LAHRPH14) is 619–625 nm, and that of the blue light (ES-CEBLM12) is 462.5–465 nm. The narrow wavelength range can not only reduce the interference from light sources but can also inhibit the deviation of Beer-Lambert’s law. Besides, the voltage-stabilizing circuit was fabricated to reduce the disturbance due to the input voltage.

The bar has been marked on the images. Both light sources were attached onto the PCB (Printed Circuit Board) and wire-bonded to sputtered Au electrodes. Subsequently, the assembly was covered with a thin layer of PDMS to protect the surface of the chip and the bonding wires.

**Figure 3:**
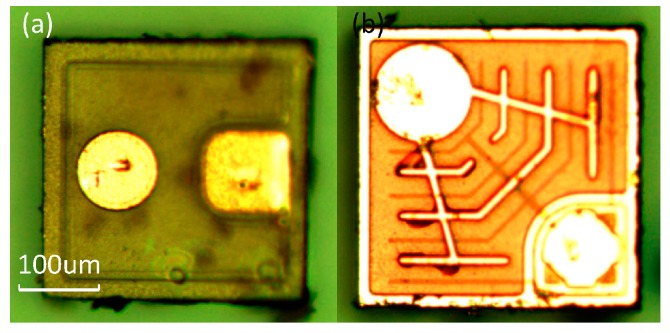
On-chip light sources. Panel (**a**) is the blue light source with a gold cathode (circular area) and anode (square area); Panel (**b**) is the red light source with a gold cathode (circular) and anode (square area).

**Figure 4:**
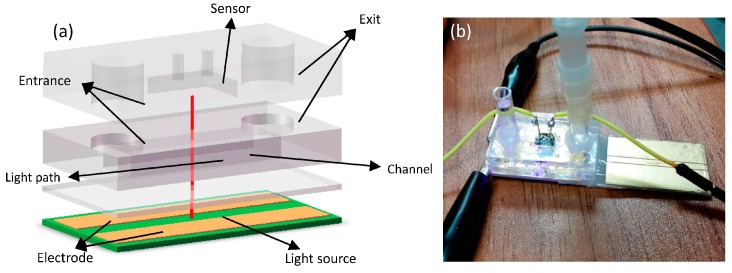
Structure of sensor and channel. Panel (**a**) depicts the sandwich structure made by PMMA, PDMS and PCB; Panel (**b**) shows the entrance connected with a peristaltic pump and tested with applied voltages.

We fabricate the structure to stabilize the sensor (s1087-01, HAMAMATSU Co., Hamamatsu, Japan) and enhance the sensitivity to some specific wavelengths of light by filtering the transmitted light. The On-chip light sources and structure of sensor are shown in Figure 3 and Figure 4 respectively.

**Figure 5:**
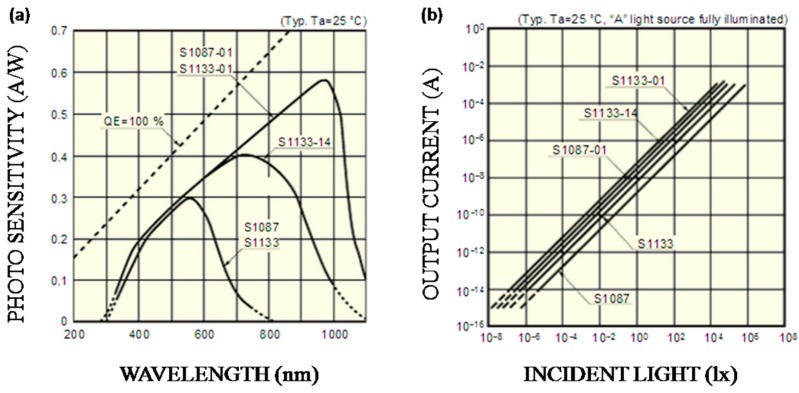
Performance of sensor (S1087-01). Panel (**a**) shows spectral response and panel (**b**) shows current linearity.

In this work, due to red light (619–625 nm) and blue light (462.5–465 nm) being selected as the light sources, S1087-01 has better photo-sensitivity than others, as Figure 5a shows. Besides, it also has a smaller volume compared to S1133-01. As Figure 5b shows, the output current of S1087-01 has a good linear relationship with the incident light intensity. Therefore, current signals have linear changes when transmitted light intensity changes; that is, electrical signals can represent the light intensity.

#### 2.2.2. Processing Circuit

**Figure 6:**
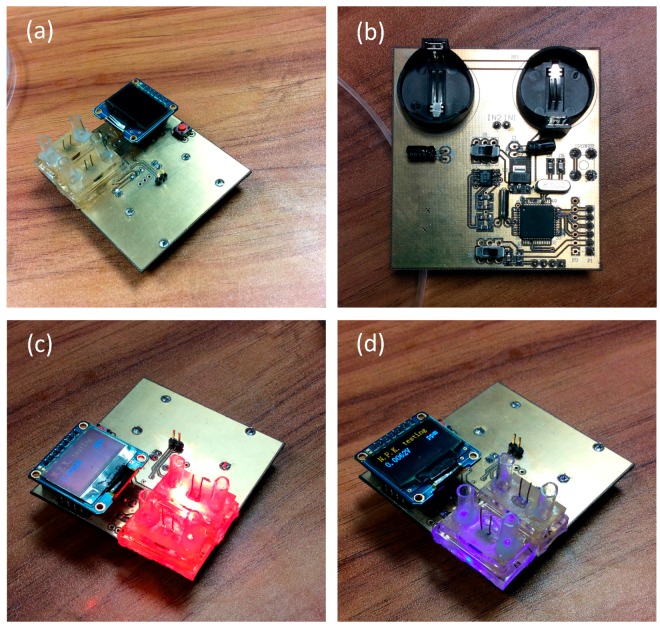
The colorimeter circuit: (**a**) top view, (**b**) bottom view, (**c**) red light working mode, and (**d**) blue light working mode.

The circuit consists of a power supply, an amplifier, a filter, a MCU (Microcontroller Unit), a displayer or a wireless transmitter. All these parts were fabricated on one chip. The code written in the MCU can be easily modified to fit more complex situations, and a mini-LED was used in this experiment to show the concentration of the solutions directly. The colorimeter circuit is shown in Figure 6.

The micro-system consists of the micro-fluidic channel, the photoelectric sensor, the signal processing module and the displayer or wireless transmission module. Photoelectric sensors (s1087-01) convert the light intensity to current signals, which is when the light intensity changes, which is caused by the changes of the solution concentrations, the current signals also change. Then, the electrical signals are amplified and filtered. Further, the MCU (F430, TI) deals with these measured signals and displays the results on the mini-LED displayer. The whole circuit can be scaled down using IC (Integrated Circuit) technology, however at an increased overall cost for low volumes. 

## 3. Sample Solution Preparation

Ammonium nitrogen preparation: we prepared our sample solutions by using standard solutions (SS); DI water (50 mL) represents 0 ppm and the standard solution (20 ppm) consists of 100 ppm ammonium nitrogen (10 mL) and KCl (40 mL). Other test solutions are prepared by diluting standard solution, as Table 1 shows. The chromogenic agent consists of phenol solution (50 mL) and NaClO (100 mL).

Available phosphorus preparation: standard solution consists of available phosphorus solution (10 mL, 100 ppm) and NaHCO_3_ (40 mL), and the chromogenic agent is Mo-Sb-Vc. Other test solutions are prepared as Table 1 shows.

Available potassium preparation: standard solution is a mixture of 100 ppm available potassium solution (10 mL) and NaNO_3_ (40 mL), and the chromogenic agent is a mixture of EDTA-2Na, formaldehyde and thymolphthalein. Other test solutions are prepared as Table 1 shows.

When we tested the anti-interference capability of the sensor, the standard samples were prepared by extracting a certain quality of real soil samples, and half of the real soil samples were extracted and worked as the tested samples.

## 4. Test Platform

The test platform consists of a micro-fluid pump (BT100-2J/YZ1515, Green Co., Beijing, China) and our colorimeter. The micro-fluid pump can control both fluid velocity and flow directions, and the flow range is from 0.002 mL/min to 380 mL/min. In our experiment, we set the revolving speed as 30 rpm to avoid the inertial focus phenomenon.

**Figure 7:**
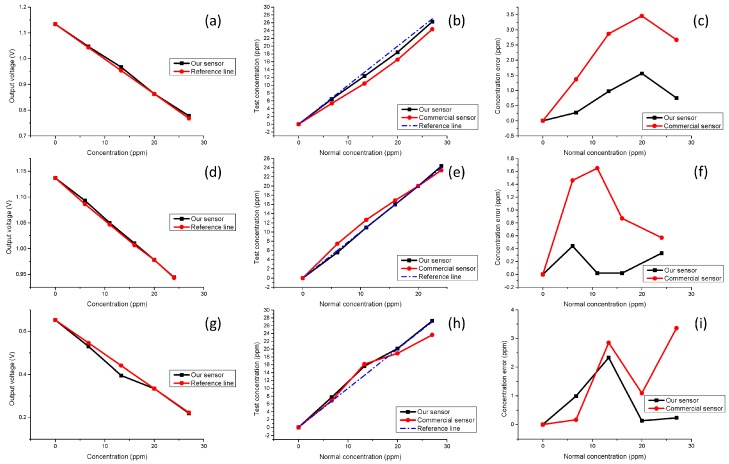
Experimental results. Panel (**a**) shows the output voltage for different concentrations of nitrogen. The back line represents test data and the red line is the reference line; Panel (**b**) shows comparison results. The blue line is the reference line which represents the normal concentration, the red line is the commercial sensor data and the black line represents our sensor; Panel (**c**) shows the detection error for different concentration of nitrogen. The red line is commercial equipment and the black line is our sensor; phosphorus and potassium test results are shown in (**d**–**f**) and (**g**–**i**), respectively.

**Figure 8:**
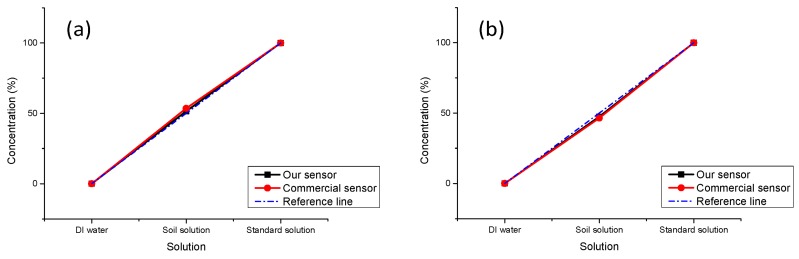
Anti-interference test results for our sensor (black line) versus the commercial equipment (red line). The blue line is the reference line. Both devices are tested in a dark environment (**a**) and in a normal light (about 300 lx) environment (**b**).

In our experiment, commercial equipment (TFC-ZNS) was chosen to make the control experiment, which was widely used in many detection areas. We prepared the solution samples and tested them. The results are shown in Figure 7 and Figure 8.

## 5. Result and Discussion

### 5.1. Control Experiments

In this experiment, we compare our detection results with the commercial ones to show the performance of our detector. 

As shown in Table 1, both the DI water and standard solution (SS) are calibration solutions, and sample solutions 1 to 4 have different concentrations and a linear relationship. All the results are measured and recorded in a dark environment. As Figure 7a,d,g show, as the NPK solution concentration increases, the transmitted light intensity becomes smaller, and the voltage signals will also decrease. Furthermore, the output voltage drops and the solution concentration has a good linear relationship for our sensor. The comparison results are shown in Figure 7b,e,h. The better performance of our sensor in terms of error control is demonstrated in Figure 7c,f,i, and the error between the test data of our sensor and the standard concentration is always smaller than that of the commercial equipment. In this experiment, due to 20 ppm standard solutions being used to calibrate concentration, our sensor shows better linearity with the concentration of the sample solutions as compared to the commercial one when testing solution 4 which is beyond the calibration range. Besides, the detection limits of our sensor are 83.6 ppm for N, 143 ppm for P, 40.9 and ppm for K, respectively.

### 5.2. Anti-Interference Capability of the System

As the sensor is based on an optical mechanism, it always has interference from ambient light; thus, a control experiment was designed to test the anti-interference capability of the system.

In this experiment, we chose soil samples from a flowerbed near our laboratory and prepared the soil solution and extracted the P elements; the detailed preparation of solutions has already been mentioned above. If we set the concentration of the standard solutions as 100%, the concentration of the soil solution should be 50%. The measurement results are shown in Figure 8. In the dark environment, our sensor gets measurement results with a smaller error (1.5%) as compared to the commercial one (3.5%). In the normal light environment, due to the interference of ambient light, greater light intensity is received by the photoelectric sensor, and the output voltage becomes lower, and the concentration which is calculated by the MCU becomes smaller. The error of our sensor is 2.5% and it is 3.5% for the commercial equipment.

During the design and experiment, there were some methods that were found to optimize the performance of the device; for example, in order to further reduce the size of the sensor or get higher sensitivity at the same size, we can optimize the path of light. We also can optimize the processing circuit by using micro-fabricated chip technology to reduce the interference and enhance SNR.

## 6. Conclusions

In this paper, a novel, portable chip-level colorimeter was fabricated to detect soil nutrients based on Beer-Lambert’s law and MEMS technology. This device exhibits a good performance in terms of error control, anti-interference capability, and linearity between output voltages and concentrations. Furthermore, the sensor performs better than commercial detectors and exhibits linearity beyond the concentration of the standard solution. The device is compact and much smaller than other colorimeters, with the whole system measuring only 6 cm × 4 cm × 6 cm. Therefore, this device can be used in soil geological exploration, experimental field detection and it can even work as a sensing node to build a monitoring network.

As the detector is based on Beer-Lambert’s law, it has a wide range of uses in many other fields. Some other mixture solutions which have high absorption rates to blue light or red light can also be detected based on the same structure, and only prepared calibration solutions and modified codes are needed. It is possible to exchange the light source to detect a larger variety of substances, e.g., by using an infrared light source, the concentration of CO_2_ can be detected and measured.

## Figures and Tables

**Table 1:** Solutions preparation list.

Solutions		DI Water	Solution 1	Solution 2	Solution 3	Solution 4	Standard Solution
SS/DI water (mL)	N element	0/50	17/33	33/17	50/0	27 ppm (50)	50/0
	P element	0/50	15/35	27.5/22.5	40/10	24 ppm (50)	50/0
	K element	0/50	17/33	33/17	50/0	27 ppm (50)	50/0
